# Supplementary material for: Barriers and Facilitators of Adherence to Nicotine Replacement Therapy: A Systematic Review and Analysis Using the Capability, Opportunity, Motivation, and Behaviour (COM-B) Model
Source: Int J Environ Res Public Health. 2020 Nov 30;17(23):8895. doi: 10.3390/ijerph17238895 (PMC7731205; doi:10.3390/ijerph17238895)
Supplement: Supplementary file 1 [file ijerph-17-08895-s001.pdf]

Supplementary material 1

Database(s): Ovid MEDLINE(R) and Epub Ahead of Print, In-Process & Other Non-Indexed Citations and Daily, 1946 to August 30, 2020
Search Strategy:

#	**Searches**
1	smoking/ or smoking reduction/ or smoking, non-tobacco products/ or tobacco smoking/
2	smoking cessation/ or "tobacco use cessation"/
3	tobacco products/ or tobacco, waterpipe/
4	patient compliance/ or medication adherence/
5	Patient Compliance/
6	"Treatment Adherence and Compliance"/
7	"tobacco use cessation devices"/ or nicotine chewing gum/
8	Nicotine replacement therapy.mp.
9	Nicotine patch.mp.
10	Nicotine inhaler.mp.
11	Lozenge.mp.
12	drug therapy/ or drug therapy, combination/
13	Medication treatment.mp.
14	1 or 2 or 3
15	4 or 5 or 6
16	7 or 8 or 9 or 10 or 11 or 12 or 13
17	14 and 15 and 16

Supplementary material 2

1. Quality assessment RCT

Questions	References													
	Berg	Cooper	De Dios	Fish	Handschin	Hood	Kim	Ojo-Fati	Okuyemi	Hollands	Vaz	Ben Taleb	Kushnir	Scherphof
Was true randomization used for assignment of participants to treatment groups?	Yes	Yes	Yes	Yes	Yes	Yes	Yes	Yes	Yes	Yes	Yes	Yes	Yes	Yes
Was allocation to treatment groups concealed?	UC	UC	Yes	Yes	Yes	Yes	Yes	Yes	Yes	UC	Yes	Yes	Yes	No
Were treatment groups similar at the baseline?	Yes	Yes	Yes	Yes	Yes	Yes	Yes	Yes	Yes	Yes	Yes	Yes	Yes	Yes
Were participants blind to treatment assignment?	Yes	UC	Yes	Yes	Yes	Yes	UC	Yes	Yes	UC	Yes	Yes	Yes	Yes
Were those delivering treatment blind to treatment assignment?	No	No	UC	UC	UC	No	UC	Yes	No	No	No	Yes	No	UC
Were outcomes assessors blind to treatment assignment?	UC	UC	UC	UC	No	No	UC	No	Yes	Yes	Yes	UC	UC	UC
Were treatment groups treated identically other than the intervention of interest?	Yes	Yes	Yes	Yes	Yes	Yes	Yes	Yes	Yes	Yes	Yes	Yes	Yes	Yes
Was follow up complete and if not, were differences between groups in terms of their follow up adequately described and analysed?	Yes	Yes	Yes	Yes	Yes	Yes	Yes	Yes	Yes	Yes	Yes	Yes	Yes	Yes
Were participants analysed in the groups to which they were randomized?	Yes	Yes	Yes	Yes	Yes	Yes	Yes	Yes	Yes	Yes	Yes	Yes	Yes	Yes
Were outcomes measured in the same way for treatment groups?	Yes	Yes	Yes	Yes	Yes	Yes	Yes	Yes	Yes	Yes	Yes	Yes	Yes	Yes
Were outcomes measured in a reliable way?	Yes	Yes	Yes	Yes	Yes	Yes	Yes	Yes	Yes	Yes	Yes	Yes	Yes	Yes
Was appropriate statistical analysis used?	Yes	Yes	Yes	Yes	Yes	Yes	Yes	Yes	Yes	Yes	Yes	Yes	Yes	Yes
Was the trial design appropriate, and any deviations from the standard RCT design (individual randomization, parallel groups) accounted for in the conduct and analysis of the trial?	UC	Yes	Yes	Yes	Yes	Yes	Yes	Yes	Yes	Yes	Yes	Yes	Yes	Yes

2. Quality assessment Cross sectional

Questions	References							
	Lam	Burns	Wiggers	Alterman	Rojewski	Shiffman	Yingst	Balmford

Were the criteria for inclusion in the sample clearly defined?	Yes	Yes	Yes	Yes	Yes	Yes	Yes	Yes
Were the study subjects and the setting described in detail?	Yes	Yes	Yes	Yes	Yes	Yes	Yes	Yes
Was the exposure measured in a valid and reliable way?	Yes	Yes	Yes	Yes	Yes	Yes	Yes	Yes
Were objective, standard criteria used for measurement of the condition?	UC	Yes	Yes	UC	Yes	UC	Yes	UC
Were confounding factors identified?	Yes	No	No	No	Yes	Yes	Yes	Yes
Were strategies to deal with confounding factors stated?	Yes	No	No	No	No	Yes	Yes	Yes
Were the outcomes measured in a valid and reliable way?	Yes	Yes	Yes	Yes	Yes	Yes	No	Yes
Was appropriate statistical analysis used?	Yes	Yes	Yes	Yes	Yes	Yes	Yes	Yes

3. Quality assessment qualitative studies

**Questions**	**References**			
	**Shadel**	**Bowker**	**Wright**	**McDaid**
Is there congruity between the stated philosophical perspective and the research methodology?	Yes	Yes	Yes	Yes
Is there congruity between the research methodology and the research question or objectives?	Yes	Yes	Yes	Yes
Is there congruity between the research methodology and the methods used to collect data?	Yes	Yes	UC	Yes
Is there congruity between the research methodology and the representation and analysis of data?	Yes	Yes	Yes	Yes
Is there congruity between the research methodology and the interpretation of results?	Yes	Yes	Yes	Yes
Is there a statement locating the researcher culturally or theoretically?	No	No	No	No
Is the influence of the researcher on the research, and vice - versa, addressed?	No	No	No	No
Are participants, and their voices, adequately represented?	Yes	Yes	Yes	Yes
Is the research ethical according to current criteria or, for recent studies, and is there evidence of ethical approval by an appropriate body?	Yes	Yes	Yes	Yes
Do the conclusions drawn in the research report flow from the analysis, or interpretation, of the data?	Yes	Yes	Yes	Yes

UC - Unclear, NA - Not applicable

PRISMA checklist

Section/topic	#	Checklist item	Reported on page #
**TITLE**			
Title	1	Identify the report as a systematic review, meta-analysis, or both.	1
**ABSTRACT**			
Structured summary	2	Provide a structured summary including, as applicable: background; objectives; data sources; study eligibility criteria, participants, and interventions; study appraisal and synthesis methods; results; limitations; conclusions and implications of key findings; systematic review registration number.	2
**INTRODUCTION**			
Rationale	3	Describe the rationale for the review in the context of what is already known.	3 and 4
Objectives	4	Provide an explicit statement of questions being addressed with reference to participants, interventions, comparisons, outcomes, and study design (PICOS).	5
**METHODS**			
Protocol and registration	5	Indicate if a review protocol exists, if and where it can be accessed (e.g., Web address), and, if available, provide registration information including registration number.	5
Eligibility criteria	6	Specify study characteristics (e.g., PICOS, length of follow-up) and report characteristics (e.g., years considered, language, publication status) used as criteria for eligibility, giving rationale.	5 and 6
Information sources	7	Describe all information sources (e.g., databases with dates of coverage, contact with study authors to identify additional studies) in the search and date last searched.	6
Search	8	Present full electronic search strategy for at least one database, including any limits used, such that it could be repeated.	6 and 7

Study selection	9	State the process for selecting studies (i.e., screening, eligibility, included in systematic review, and, if applicable, included in the meta-analysis).	6
Data collection process	10	Describe method of data extraction from reports (e.g., piloted forms, independently, in duplicate) and any processes for obtaining and confirming data from investigators.	7
Data items	11	List and define all variables for which data were sought (e.g., PICOS, funding sources) and any assumptions and simplifications made.	5 and 6
Risk of bias in individual studies	12	Describe methods used for assessing risk of bias of individual studies (including specification of whether this was done at the study or outcome level), and how this information is to be used in any data synthesis.	7
Summary measures	13	State the principal summary measures (e.g., risk ratio, difference in means).	N/A
Synthesis of results	14	Describe the methods of handling data and combining results of studies, if done, including measures of consistency (e.g., I^2) for each meta-analysis.	9

Page 1 of 2

**Section/topic**	**#**	**Checklist item**	**Reported on page #**
Risk of bias across studies	15	Specify any assessment of risk of bias that may affect the cumulative evidence (e.g., publication bias, selective reporting within studies).	7
Additional analyses	16	Describe methods of additional analyses (e.g., sensitivity or subgroup analyses, meta-regression), if done, indicating which were pre-specified.	N/A
**RESULTS**			
Study selection	17	Give numbers of studies screened, assessed for eligibility, and included in the review, with reasons for exclusions at each stage, ideally with a flow diagram.	9 and 10

Study characteristics	18	For each study, present characteristics for which data were extracted (e.g., study size, PICOS, follow-up period) and provide the citations.	10 and 11
Risk of bias within studies	19	Present data on risk of bias of each study and, if available, any outcome level assessment (see item 12).	7
Results of individual studies	20	For all outcomes considered (benefits or harms), present, for each study: (a) simple summary data for each intervention group (b) effect estimates and confidence intervals, ideally with a forest plot.	N/A
Synthesis of results	21	Present results of each meta-analysis done, including confidence intervals and measures of consistency.	12 - 22
Risk of bias across studies	22	Present results of any assessment of risk of bias across studies (see Item 15).	7
Additional analysis	23	Give results of additional analyses, if done (e.g., sensitivity or subgroup analyses, meta-regression [see Item 16]).	N/A
**DISCUSSION**			
Summary of evidence	24	Summarize the main findings including the strength of evidence for each main outcome; consider their relevance to key groups (e.g., healthcare providers, users, and policy makers).	22-25
Limitations	25	Discuss limitations at study and outcome level (e.g., risk of bias), and at review-level (e.g., incomplete retrieval of identified research, reporting bias).	26
Conclusions	26	Provide a general interpretation of the results in the context of other evidence, and implications for future research.	27
**FUNDING**			
Funding	27	Describe sources of funding for the systematic review and other support (e.g., supply of data); role of funders for the systematic review.	27

From: Moher D, Liberati A, Tetzlaff J, Altman DG, The PRISMA Group (2009). Preferred Reporting Items for Systematic Reviews and Meta-Analyses: The PRISMA Statement. PLoS Med 6(7): e1000097. doi:10.1371/journal.pmed1000097

For more information, visit: **www.prisma-statement.org**.
